# The Mink Circovirus Capsid Subunit Expressed by Recombinant Baculovirus Protects Minks against Refractory Diarrhea in Field

**DOI:** 10.3390/v13040606

**Published:** 2021-04-01

**Authors:** Lidong Wang, Yanyan Zhang, Teng Chen, Lijuan Mi, Xuefei Sun, Xintao Zhou, Faming Miao, Shoufeng Zhang, Ye Liu, Rongliang Hu

**Affiliations:** Laboratory of Epidemiology, Institute of Military Veterinary Medicine, Academy of Military Medical Sciences, Academy of Military Sciences, Jilin 130122, China; 15164390097@163.com (L.W.); Yanyanzhang90615@163.com (Y.Z.); ctcx1991@163.com (T.C.); mlj84321@163.com (L.M.); sunxuefei0322@163.com (X.S.); zhouxtao@foxmail.com (X.Z.); miaofaming81@163.com (F.M.); zhangshoufeng@hotmail.com (S.Z.); liuye79@126.com (Y.L.)

**Keywords:** mink circovirus, capsid subunit, recombinant baculovirus, field vaccination

## Abstract

Mink refractory diarrhea is a seasonal disease that occurs in many mink farms in China. Mink circovirus (MiCV) has been recognized as the causative agent of the disease. The aim of the study was to develop a subunit vaccine against mink refractory diarrhea. A recombinant baculovirus strain expressing the capsid protein was constructed using the baculovirus expression vector system (BEVS). A subunit vaccine was developed based on the capsid protein with appropriate adjuvant. Then, a field trial was carried out in two districts in order to evaluate the efficiency of the subunit vaccine. The field trial indicated that in total, only 1.8% of the minks developed typical diarrhea in the vaccinated group compared with 74.5% in the control group. The vaccination could significantly reduce the infection rate of MiCV among the mink herds and could restrain the virus’ shedding from feces. Furthermore, the vaccinated group had a higher average litter size in the following year compared to the control group. Collectively, the results indicated that the subunit vaccine based on the capsid protein can provide reliable protection against MiCV infection.

## 1. Introduction

Mink refractory diarrhea, also called “autumn diarrhea” or “red and grey diarrhea” by local farmers, has appeared in mink farms in China since the mid-1980s. In a typical outbreak, usually 70–80% of minks on a farm develop diarrhea, anorexia, and poor fur quality, and 7−8% die as a result [1]. Due to the considerable economic loss caused by the disease, an autogenous vaccine, which was made using formalin-inactivated gut tissue suspension of the infected minks, was initially put into application. This strategy indeed reduced the morbidity and partially controlled the disease for a period of time around the 2000s. However, after a few years of application, the vaccinated pedigree breeds appeared to show symptoms of Aleutian mink disease. Given the high infection rate of the Aleutian mink disease virus ( >60%) among mink herds in China [2], it can be speculated that the autogenous vaccine contains inactivated Aleutian mink disease viruses that enhance immune complex formation and lead to the Aleutian mink disease [3]. Hence, the autogenous vaccine has been abandoned in recent years, and there is an urgent need for a safe and efficient vaccine to control the disease.

Mink circovirus (MiCV) was first discovered in mink farms in Dalian, China, in 2013 and has been recognized as the etiological agent of mink refractory diarrhea [1,4]. MiCV belongs to the genus *circovirus* of the family *Circoviridae* and it is composed of a capsid protein and a circular, single-stranded genome that contains two open reading frames (ORFs), named ORF1 (rep gene, 894 bp) and ORF2 (cap gene, 684 bp) [5]. Phylogenetic analysis of the cap gene showed that MiCV was genetically closest to the bat circovirus, with a sequence similarity of about 73% [6]. For the members of *circovirus*, the capsid protein is usually considered to play a key role in the immunoprotection against infection. For instance, one dose of a porcine circovirus type 2 subunit vaccine based on the capsid protein demonstrated the ability to induce both a humoral and cellular immune response, thus reducing the morbidity, mortality rate, viremia, and viral load under the field trial [7].

The baculovirus expression vector system (BEVS) is a kind of eukaryotic expression system. Since it was initially used for the production of human β-interferon successfully in 1983 [8], BEVS made a great contribution to the development of veterinary subunit vaccines. The first globally available commercial veterinary vaccine based on BEVS was the E2 subunit vaccine against classical swine fever. Two weeks after vaccination, pigs could significantly resist against the intranasal challenge with 100 median lethal dose (LD_50_) of the virulent classical swine fever virus [9]. The above-mentioned porcine circovirus type 2 subunit vaccine is also based on the capsid protein expressed by BEVS. Furthermore, some other vaccines such as bluetongue virus vaccines [10,11,12] and avian influenza virus vaccines [13,14] have also shown good immunogenicity, despite having no commercial application at present.

In this study, we aimed to utilize the BEVS to express the capsid protein of MiCV and develop a subunit vaccine against mink refractory diarrhea. Subsequently, we carried out a field trial in mink farms. The efficiency of the subunit vaccine was evaluated by a clinical symptom observation, fecal sample test, and average litter size analysis.

## 2. Materials and Methods

### 2.1. Generation and Production of Recombinant Baculoviruses

A Bac-to-Bac baculovirus expression system (Life Technologies, Carlsbad, CA, USA) was used for the generation and production of recombinant baculoviruses. MiCV-DL13 (an MiCV strain first discovered in Dalian, China, in 2013) (NC_023885.1) samples were derived from the livers and intestines of infected minks and were kept frozen at −80 °C. DNA was extracted from the MiCV samples using a viral DNA extraction kit (Corning, Wujiang, China). The capsid gene was cloned using a polymerase chain reaction (PCR) approach with primers containing homologous ends of the pFastBacI transfer plasmid (forward: 5’-CGCGGCCGCTTTCGAATCTAGAATGCCCGTAAGATCGCGATACT−3’, and reverse: 5’-ACTTCTCGACAAGCTTGGTACCTTAGTGATGGTGATGGTGATGAGTTTGCTTTGGGAAATTG−3’—the underlined sequences at 5′ were the homologous ends of the pFastBacI transfer plasmid and the underlined sequence in the middle of the reverse primer was the sequence of the 6 × His fusion tag). The pFastBacI vector was linearized using restriction enzymes (*Xba I* and *Kpn I*) (New England Biolabs, Beijing, China) and the encoding sequence of the capsid protein with the C-terminal 6 × His fusion tag was seamlessly inserted via external homologous recombination with an in-fusion cloning kit (Takara, Beijing, China) following the manufacturer’s protocol. The recombinant clones were transferred into *Escherichia coli* DH5α (Takara, Beijing, China) for amplification and were extracted with a plasmid miniprep kit (Corning, Wujiang, China). The recombinant plasmids were verified using PCR with pFastBacI vector primers (forward: 5’-CTCCGGAATATTAATAGATC−3’, and reverse: 5’-CAAATGTGGTATGGCTGATT−3’). The recombinant transfer vectors were sequenced to ensure that no mutation occurred.

The recombinant transfer vectors were transformed into *Escherichia coli* DH10Bac competent cells (Biomed, Beijing, China) and incubated on agar plates containing 50 μg/mL kanamycin, 7 μg/mL gentamicin, 10 μg/mL tetracycline, 100 μg/mL 5-Bromo−4-chloro−3-indolyl β-D-galactopyranoside (X-gal), and 40 μg/mL isopropyl-beta-D-thiogalactopyranoside (IPTG) (Takara, Beijing, China) for 48 h at 37 °C. The white clones were selected and amplified in liquid culture containing 50 μg/mL kanamycin, 7 μg/mL gentamicin, and 10 μg/mL tetracycline. Then, the recombinant bacmids were extracted using alkaline lysis, described elsewhere [15], from the bacteria and were further verified using PCR with M13 primers (forward: 5’-CCCAGTCACGACGTTGTAAAACG−3’, and reverse: 5’-AGCGGATAACAATTTCACACAGG−3’). The naked transfer vectors were transformed into *Escherichia coli* DH10Bac competent cells in the same way.

Sf9 cells (Life Technologies, Carlsbad, CA, USA) were transfected with recombinant bacmids. Confluency and viability were assessed using a trypan blue staining cell viability assay kit (Beyotime Biotechnology, Shanghai, China) following the manufacturer’s instruction manual. Briefly, 2 μg recombinant bacmids and 6 μL transfection reagent (Mirus Bio, Madison, WI, USA) were incubated in 100 µL Sf-900 II serum-free medium (Life technologies, Carlsbad, CA, USA) for about 5 min each and mixed gently. The mixture was incubated at room temperature for 20 min and was added dropwise to the culture of monolayer Sf9 cells at 70% confluency. The transfected cells were cultured in Sf-900 II serum-free medium with 10% heat-inactivated fetal calf serum at 27 °C in a cabinet for 5 days to generate initial recombinant baculoviruses. The non-recombinant baculoviruses were generated from naked bacmids in the same way as the control.

### 2.2. Expression of the Capsid Protein

Sf9 cells at 90% confluency were infected with recombinant baculoviruses at a multiplicity of infection (MOI) of 0.1 and incubated in the dark at 27 °C for the expression of capsid protein. Furthermore, High Five (H5) cells (Life Technologies, Carlsbad, CA, USA) were seeded by recombinant baculoviruses at the same MOI and were cultured in suspension in Sf-900 II serum-free medium at 27 °C at the speed of 110 rpm/min for 72 h for rapid production of the capsid protein. In addition, Sf9 cells and H5 cells were both infected with non-recombinant baculoviruses in the same way as the control.

### 2.3. PCR Identification of Recombinant Baculoviruses

The H5 cells were harvested 72 h post-infection (pi). Then, genomic DNA was extracted from the cells using a viral DNA miniprep kit (Corning, Wujiang, China), following the manufacturer’s instruction manual, and was identified using PCR with M13 primers (forward: 5′−CCCAGTCACGACGTTGTAAAACG−3′, and reverse: 5′−AGCGGATAACAATTTCACACAGG−3′) to verify the presence of the recombinant baculoviruses.

### 2.4. Indirect Immunofluorescence Assay

The Sf9 cell monolayer was gently washed twice with phosphate-buffered saline (PBS) (pH = 7.4) and fixed with 80% cold acetone for 30 min at 4 °C. Two kinds of antibodies, anti-His tag monoclonal antibody (CWBIO, Beijing, China) and tetramethylrhodamine isothiocyanate (TRITC)-conjugated goat anti-mouse immunoglobulin G (IgG) (CWBIO, Beijing, China), were used for an incubation with the cell monolayer for 1 h at 37 °C successively. Then, the cell monolayer was washed three times with PBS with 0.05% Tween-20 (PBST) and was watched under a florescent microscope.

### 2.5. Sodium Dodecyl Sulfate-Polyacrylamide Gel Electrophoresis (SDS-PAGE) and Western Blot Analysis

The H5 cells were harvested 72 h pi and were washed three times with PBS, counted, and resuspended in PBS at a concentration of 2 × 10^6^ cell equivalents per milliliter. The cells were frozen and thawed twice for lysis. The crude lysate was mixed with a sample buffer and boiled for 5 min. After a simple centrifugation, the proteins in the supernatant were separated on polyacrylamide gel. After that, the polyacrylamide gel was stained using Coomassie brilliant blue solution for 2 h and was then de-stained for several hours for observation. In addition, another same polyacrylamide gel was transferred onto a polyvinylidene fluoride (PVDF) membrane (Bio-Rad, Berkeley, CA, USA). The membrane was blocked with 5% skim milk in PBS at 4 °C overnight and probed with an anti-His tag monoclonal antibody (CWBIO, Beijing, China). Then, the membrane was probed with a horseradish peroxidase (HRP)-conjugated goat anti-mouse IgG (CWBIO, Beijing, China). After being washed with PBST three times, the membrane was developed using tetramethylbenzidine (TMB) (Kirkegaard & Perry Laboratories, Gaithersburg, MD, USA), a horseradish peroxidase substrate, according to the manufacturer’s instructions.

### 2.6. Vaccine Preparation

The H5 cells were harvested 72 h pi, counted, and prepared at a concentration of 2 × 10^6^ cell equivalents per milliliter of cell culture. The cells were frozen and thawed twice for lysis. Then, 0.9 mL crude lysate from H5 cells infected by recombinant baculoviruses and 0.1 mL 10% aluminum hydroxide gel (Thermo Fisher Scientific, Waltham, MA, USA) were fully mixed and stirred as one dose of subunit vaccine. For the control, 0.9 mL pure cell culture was mixed under the same condition. All of the preparations were stored on ice until inoculation.

### 2.7. Animal Experimental Design

We performed a field vaccination trial in selected mink farms in Zhuanghe, Liaoning, and Zhucheng, Shandong. The animals included in the trial were American minks (*Neovison vison*) (half male and half female) at about 4 months of age. The field trial began at the end of June in Zhuanghe, Liaoning, and at the end of August in Zhucheng, Shandong. In total, 2000 animals received one dose of the subunit vaccine (1 mL) intramuscularly as the vaccinated group (Vaccinated). Meanwhile, another 1000 animals received the equivalent control preparation (cell culture mixed with aluminum hydroxide gel) in the same way as the control group (Control). The experimental unit was a single mink in a single cage. Housing, husbandry, feeding, and other conditions were kept identical for both experimental groups in each district. The operators and keepers were blind to the experimental design. Table 1 lists the number of animals included in each group and in each district.

About two or three months after vaccination, from the end of September to the end of November, the most common time for mink refractory diarrhea, the two groups were clinically monitored daily for typical diarrhea.

Seventy fecal samples from each group in each district were randomly collected. The samples were fully mixed with tris (hydroxymethyl) aminomethane (Tris)-buffered saline and were centrifuged at a speed of 5000 r/min for 15 min. The viral DNA in the supernatant was extracted using a viral DNA extraction kit (Corning, Wujiang, China) and was detected using PCR with primer pairs described elsewhere [16].

Around late April to early May of the following year, when the whelping season came, the number of pregnant minks and their offspring was recorded. Then, the average litter size of each group was calculated in order to evaluate the fecundity. The average litter size usually means the ratio of newborn offspring to previous pregnant animals in a group.

### 2.8. Statistical Analysis

The data of morbidity among mink herds and positive rate of the fecal samples were analyzed using the Pearson chi-square test. The data of the average litter size are presented as mean ± SD values. Statistical analysis of the average litter size was performed by applying Student’s *t*-test. *p*-values < 0.05 were considered statistically significant. The statistical analysis was performed using IBM SPSS Statistics software (ver.19.0; IBM, Armonk, NY, USA).

## 3. Results

### 3.1. The Capsid Protein of MiCV Was Successfully Expressed

**Figure 1:**
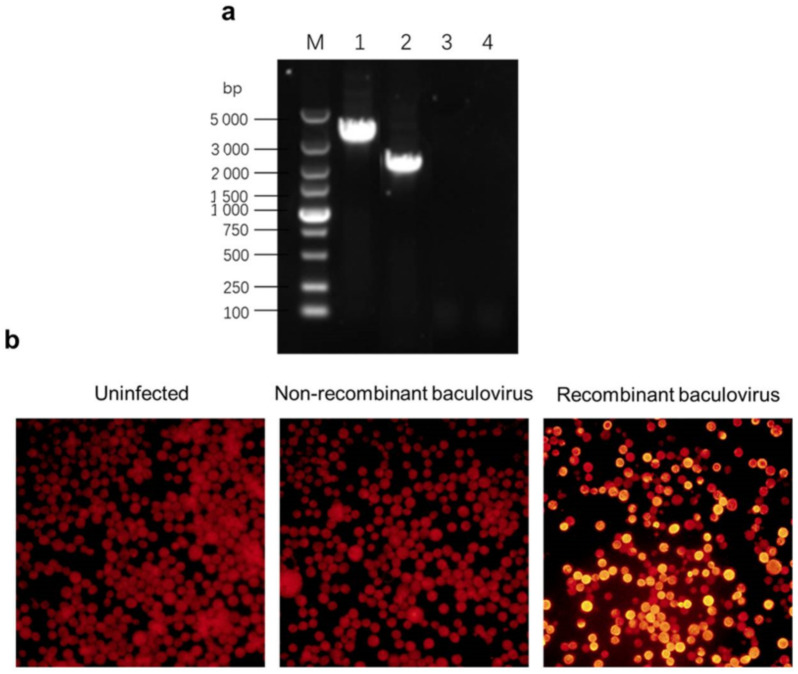
Agarose gel electrophoresis and fluorescence imaging analyses of recombinant baculoviruses. (**a**) Agarose gel electrophoresis analysis. Lane 1: Polymerase chain reaction (PCR) product of recombinant baculoviruses; lane 2: PCR product of non-recombinant baculoviruses; lane 3: PCR product of uninfected H5 cells; lane 4: negative control. (**b**) Fluorescence imaging analysis. Sf9 cells that were infected by non-recombinant or recombinant baculoviruses were detected under a fluorescent microscope. The stain was tetramethylrhodamine isothiocyanate (TRITC) and the original magnification was 100×.

The recombinant baculoviruses were confirmed by PCR (Figure 1a). The indirect immunofluorescence assay showed that a specific bright orange–red image was captured in the Sf9 cells infected by recombinant baculoviruses, while there was no such image in the Sf9 cells infected by non-recombinant baculoviruses, nor in the uninfected cells (Figure 1b). (The results of identification of recombinant transfer vectors and bacmids are shown in Supplementary Figure S1.)

**Figure 2:**
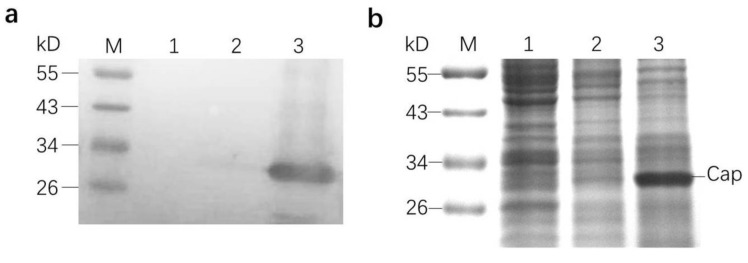
SDS-PAGE and Western blot analyses of the capsid protein. (**a**) Western blot analysis. (**b**) SDS-PAGE analysis showed the molecular weights of the capsid protein. Lane 1: Protein from crude lysate of uninfected H5 cells; lane 2: protein from crude lysate of H5 cells infected by non-recombinant baculoviruses; lane 3: protein from crude lysate of H5 cells infected by recombinant baculoviruses. Cap: capsid protein.

The Western blot analysis confirmed the presence of the capsid protein (Figure 2a). The SDS-PAGE analysis indicated that the molecular weight of the capsid protein was about 27 kDa and the amount of capsid protein was obviously larger than that of other irrelevant proteins (Figure 2b).

### 3.2. Reduction in Morbidity and Infection Rate in Vaccinated Animals

**Figure 3:**
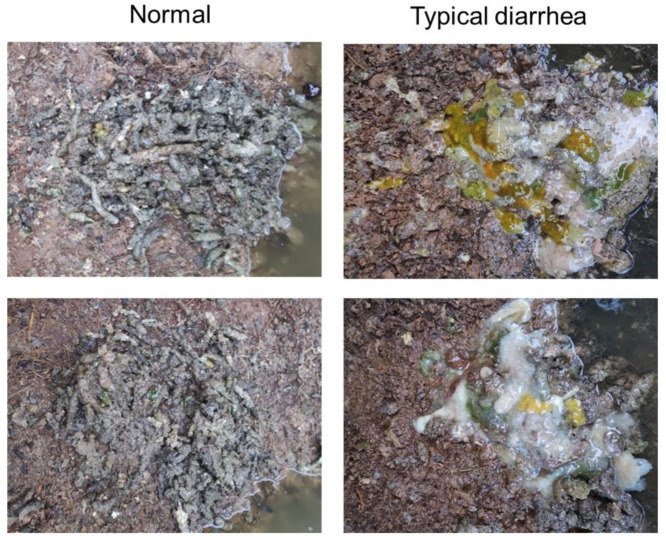
Appearances of normal feces and typical diarrhea. Two samples are displayed for each category. The feces of healthy minks were dark green and relatively dry (normal), while those of sick minks were yellow, grey, and waterish (typical diarrhea), which is the classical symptom of mink refractory diarrhea.

**Figure 4:**
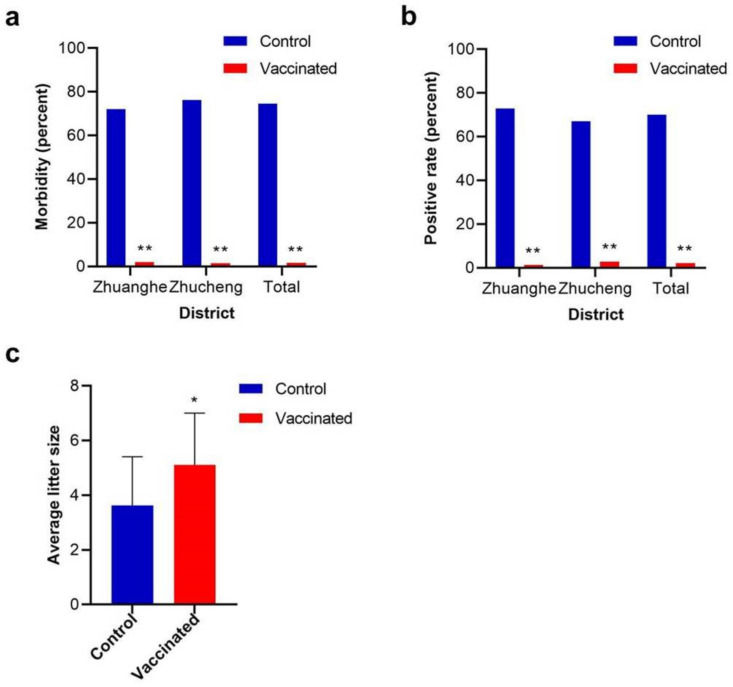
Comparison of morbidities, positive rates of fecal samples, and average litter sizes between control and vaccinated groups. (**a**) Comparison of morbidities between control and vaccinated groups in Zhuanghe, Zhucheng, and total. (**b**) Comparison of positive rates of fecal samples between control and vaccinated groups in Zhuanghe, Zhucheng, and total. (**c**) Comparison of average litter sizes between control and vaccinated groups in total (* *p* < 0.05; ** *p* < 0.01).

According to the observation of feces (the appearances of normal feces and typical diarrhea are shown in Figure 3), in total, only 36/2000 (1.8%) minks developed typical diarrhea in the vaccinated groups compared to 745/1000 (74.5%) in the control groups. The morbidity rates among the vaccinated groups were far lower than those of the control groups regardless of district or in total (Figure 4a).

The PCR test of fecal samples showed that the infection rate of MiCV among the vaccinated groups was 2.1%, while that of the control groups was 70.0%. The infection rates among the vaccinated groups were obviously lower than those of the control groups regardless of district or in total (Figure 4b).

### 3.3. Comparasion of Average Litter Size

In total, 500 and 250 pregnant minks in the vaccinated and control groups were observed, respectively. The average litter size of the vaccinated groups in the following year was 5.11 ± 1.89, while that of the control groups was 3.62 ± 1.78 (Figure 4c). The vaccinated group showed a higher fecundity.

## 4. Discussion

Circovirus has various hosts among mammals and birds. In addition to the well-known porcine circovirus, circoviruses that can infect canines, penguins, bats, and other animals have been reported [17,18,19] in recent years. As a new circovirus member, MiCV has only been discovered in China and was even reported to be able to infect foxes and raccoon dogs [5]. Although MiCV can cause relatively large economic loss to the mink industry, few valid prevention technologies are available at present. Some new diagnostic techniques such as real-time quantitative PCR, indirect enzyme-linked immunosorbent assay, and recombinase polymerase amplification have been established [20,21,22]. However, vaccines against MiCV have not yet been developed. In this study, the subunit vaccine against MiCV was confirmed to be effective.

The capsid protein of MiCV has been previously expressed in the yeast system by the researchers in our laboratory [23]; nonetheless, the yield was too limited (less than 20 µg/mL) for vaccine development. The potential for high-level recombinant protein production is one of the advantages of the BEVS, with “high-level” defined rather loosely as ≥100 μg of recombinant protein per milliliter of infected insect cell culture [24]. In this study, the highest yield of the capsid protein reached 150 μg/mL.

Up to now, MiCV has not been successfully cultured in any cell lines yet. Therefore, it is a challenge to evaluate the efficacy of vaccines considering the lack of efficient artificial infection methods. However, mink refractory diarrhea is a typical seasonal disease with classic symptoms that can be easily recognized. Furthermore, a previous investigation indicated that the disease occurs almost every year at a regular time, with relatively stable morbidity [1]. Therefore, we made the judgement that natural infection is a feasible method for the field trial. According to the experience of local farmers, there is a close relationship between the epidemic time and low temperature. This is the reason why the field trial started at different times in two districts. The infection rate of MiCV in mink farms in China was approximately 54.6% in 2014 [6], while in this study, the infection rate among control groups reached nearly 70%, partially because the animals included in the field trial were all newborn in April or May of that year. Using younger animals avoided the potential influence of original antibodies because these animals had little chance of being infected by MiCV before the field trial. Given the instability of natural infection, a large number of animals were included in the field trial in order to obtain reliable data. However, as no artificial infection was included, the field trial did not inflict much additional suffering on the animals.

MiCV was reported to be able to cause a reduction in litter size and smaller newborn cubs [25]. In this study, we indeed confirmed this phenomenon. Usually, the highest average litter size of mink herds in China is about 5.50, and that of the vaccinated group in this study essentially reached the normal level, but 3.62 in the control group was, no doubt, a significant decline. Some other circoviruses, such as porcine circovirus type 2, can inflict reproductive failure, including abortions, stillbirths, etc. [26]. However, in addition to purely low litter size, we did not observe further reproductive failure symptoms as seen in porcine circovirus type 2. The mechanism is poorly understood at present.

During the field trial, no adverse effect of the vaccination was observed, which means that the vaccine causes no harm to the body. Due to the lack of a strict challenge experiment, the minimum dose of the vaccine has not been ascertained yet. In addition, the dose–effect relationship, the antibody titer, and other evaluation indexes of the subunit vaccine call for further research.

In summary, the capsid protein subunit vaccine was successfully constructed with BEVS and showed expectative effectiveness and safety. The field trial indicated that the subunit vaccine can reduce the morbidity, restrain the viruses shedding from the feces, and improve the fecundity. The subunit vaccine is expected to become a reliable biological product to contain MiCV infection in China.

## Figures and Tables

**Table 1:** The number of animals included in each group and each district.

District	Vaccinated	Control	Total
Zhuanghe	800	400	1200
Zhucheng	1200	600	1800
Total	2000	1000	3000

## Data Availability

Not applicable.

## Supplementary Materials

The following are available online at https://www.mdpi.com/article/10.3390/v13040606/s1, Figure S1: Identification of transfer vectors and recombinant bacmids.
