# Supplementary material for: The Mink Circovirus Capsid Subunit Expressed by Recombinant Baculovirus Protects Minks against Refractory Diarrhea in Field
Source: Viruses. 2021 Apr 1;13(4):606. doi: 10.3390/v13040606 (PMC8066883; doi:10.3390/v13040606)
Supplement: Supplementary file 1 [file viruses-13-00606-s001.pdf]

Supplementary Information

Supplementary Figures:

Figure S1

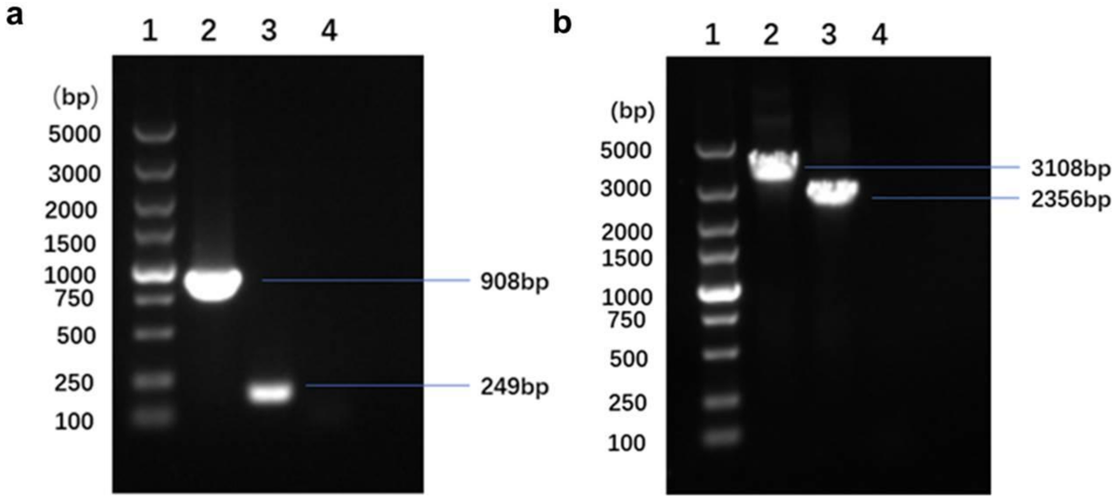

Figure S1. Identification of recombinant transfer vectors and recombinant bacmids. (a) Identification of recombinant transfer vectors by PCR. Lane 1: 5000bp DNA Marker; lane 2: recombinant transfer plasmid; lane 3: naked transfer plasmid; lane 4: negative control. (b) Identification of recombinant bacmids by PCR. Lane 1: 5000 bp DNA Marker; lane 2: recombinant bacmids; lane 3: naked bacmids; lane 4: negative control.
